# Prognostic Significance of Hemoglobin, Albumin, Lymphocyte, and Platelet (HALP) Score in Liver Transplantation for Hepatocellular Carcinoma

**DOI:** 10.3390/curroncol32080464

**Published:** 2025-08-16

**Authors:** Imam Bakir Bati, Umut Tuysuz, Elif Eygi

**Affiliations:** 1 Department of Liver Transplant Surgery, Faculty of Medicine, Acıbadem University, Istanbul 34752, Turkey; imam.bati@acibadem.com; 2 Department of Liver Transplant Surgery, Şişli Etfal Hamidiye Training and Research Hospital, Istanbul 34360, Turkey; umutuysuz@gmail.com; 3 Department of Anesthesiology and Reanimation, Gaziantep City Hospital, Gaziantep 27100, Turkey

**Keywords:** hepatocellular carcinoma, liver transplantation, HALP score, GPR, FIB-4, overall survival, recurrence-free survival, prognostic biomarker

## Abstract

*Objectives:* Hepatocellular carcinoma (HCC) remains a major indication for liver transplantation (LT), but accurate pretransplant risk stratification is critical to improve long-term outcomes. Traditional morphometric criteria such as tumor size and number are limited in predicting recurrence and survival. The HALP (hemoglobin, albumin, lymphocyte, platelet), gamma-glutamyl transpeptidase to platelet ratio (GPR), and FIB-4 indices are emerging systemic inflammatory and nutritional biomarkers that may provide additional prognostic value in HCC patients undergoing LT. *Materials and Methods:* This retrospective, two-center cohort study included 200 patients who underwent LT for HCC between 2012 and 2023. Preoperative HALP, GPR, and FIB-4 scores were calculated, and their associations with overall survival (OS) and recurrence-free survival (RFS) were assessed using ROC analyses and Cox proportional hazard models. Cut-off values were determined for each biomarker, and survival outcomes were analyzed using Kaplan–Meier methods. *Results:* A low HALP score (≤0.39) was independently associated with reduced OS but not with RFS. Conversely, low GPR (≤0.45) and FIB-4 (≤3.1) values were significantly associated with both poor OS and higher recurrence risk. Tumor size, number of lesions, and microvascular invasion also independently predicted poor outcomes. Multivariate analysis confirmed HALP, GPR, and FIB-4 as significant preoperative predictors of prognosis in this population. *Conclusions:* HALP, GPR, and FIB-4 are readily available, cost-effective indices that provide significant prognostic information in HCC patients undergoing LT. Their integration with morphometric criteria may improve pretransplant risk stratification and support individualized clinical decision-making.

## Simple Summary

Liver cancer, specifically hepatocellular carcinoma, is one of the most common and deadly cancers worldwide. For many patients, liver transplantation offers the best chance of long-term survival, as it replaces both the diseased liver and removes the cancer. However, predicting which patients will live longer and have a lower risk of the cancer coming back after transplantation is challenging. In this study, we looked at three simple blood-based scores that reflect a person’s nutritional health, immune function, and liver condition: the HALP score (based on hemoglobin, albumin, lymphocyte count, and platelet count), the FIB-4 index (based on age and common liver blood tests), and the GPR ratio (based on a liver enzyme test and platelet count). We analyzed information from 200 patients who had a liver transplant for liver cancer. We found that these scores, measured before the operation, could help identify patients at higher risk of poor survival or cancer returning after surgery. Because these tests are easy to perform, inexpensive, and available in most hospitals, they could be used alongside existing medical criteria to improve patient selection and tailor follow-up care after liver transplantation.

## 1. Introduction

Hepatocellular carcinoma is the seventh most prevalent cancer globally and the third leading cause of cancer-related mortality [1,2,3]. In cirrhotic livers, there are options for liver resection, locoregional treatment, and systemic therapy for hepatocellular carcinoma. While liver resection is an effective option in early-stage cancer, the high risk of recurrence hampers its preference. In addition, Major hepatectomy performed in patients with cirrhosis is associated with a significant risk of complications and death [3,4]. Some tumor localizations often pose barriers to resection. However, the only curative treatment that eliminates both cirrhotic liver and cancer is liver transplantation. Cirrhosis is a major risk factor for new lesion occurrence. Liver transplantation provides excellent post-transplant outcomes with a low risk of hepatocellular carcinoma recurrence, especially when strict and meticulous patient selection is performed.

Approximately 20–40% of liver transplants worldwide are performed due to hepatocellular carcinoma [5]. Hepatocellular carcinoma recurrence plays the most crucial role in post-transplantation prognosis. Therefore, various criteria and grading systems have been established to identify candidates who will benefit maximally from transplantation to achieve good outcomes. One of the aims here is to target rates that can achieve comparable long-term results to liver transplants performed for benign indications (at least a 50% total survival cut-off value at 5 years) [6].

In recent decades, it has been reported that inflammation related to cancer plays a significant role in cancer development. It is known to be important in tumorigenesis within the hepatocellular microenvironment. Particularly, the immune microenvironment is crucial in influencing tumor development and patient outcomes. In parallel, the immune structure of hepatocellular carcinoma is closely associated with treatment outcomes and survival. Many inflammatory markers have also been proven to be prognostic factors in hepatocellular carcinoma [7].

Many inflammatory markers have been proven to be prognostic factors in hepatocellular carcinoma [8]. The combination of these inflammatory markers has led to the development of the HALP (hemoglobin, albumin, lymphocyte, platelet) scoring model, which has a high predictive capability for the prognosis of various tumors such as pancreatic cancer, gastrointestinal stromal tumors, and esophageal cancer [9]. Despite effective outcomes reported for hepatocellular carcinoma undergoing liver resection, no study has yet been conducted to determine the overall survival, recurrence risk, and prognosis in hepatocellular carcinoma patients after liver transplantation. Additionally, indices such as the gamma-glutamyl transpeptidase to platelet ratio (GPR) and fibrosis-4 (FIB-4) reflect the degree of inflammation and fibrosis in the liver [10,11]. While GPR is a prognostic marker used in various tumors, its importance in hepatocellular carcinoma remains a topic of debate. Consequently, the relationship between HCC prognosis and GPR has garnered significant interest [12]. Furthermore, many studies have reported that the FIB-4 index in cirrhotic livers has a significant impact on overall survival in patients with hepatocellular carcinoma [13].

Recent advancements in prognostic modeling further underscore the growing importance of predictive biomarkers in HCC transplantation. In a large international cohort, the 2025-developed TRIUMPH model demonstrated superior accuracy in predicting post-transplant recurrence compared with traditional biomarker-including models [14,15]. Similarly, enhancing the RETREAT score with AFP-L3 and DCP (mRETREAT) significantly improved discriminatory power for recurrence risk (AUC 0.86 vs. 0.82) [16]. Additionally, emerging transplantation techniques, such as machine perfusion, have shown improved recurrence-free survival compared to static cold storage [17], highlighting the evolving landscape in transplant oncology.

In light of all these considerations, the present study primarily aimed to investigate the association between the preoperative HALP score and key post-transplant outcomes—namely recurrence risk, OS, and prognosis—in patients with hepatocellular carcinoma undergoing liver transplantation. Recognizing that traditional morphometric criteria alone may not fully capture biological tumor behavior, our secondary objective was to evaluate the prognostic predictive ability of two additional, non-invasive indices: the FIB-4 score and the GPR. By integrating these systemic inflammatory and immunonutritional markers with established clinical and pathological parameters, we sought to determine whether a multimodal risk stratification approach could improve patient selection, refine prognostic assessment, and ultimately contribute to individualized treatment planning in modern transplant oncology.

## 2. Materials and Methods

### 2.1. Study Design

This retrospective, observational cohort study was conducted at two high-volume liver transplantation centers in Turkey and included adult patients who underwent liver transplantation (LT) for hepatocellular carcinoma (HCC) between January 2012 and December 2023. This study was approved by the ethics committee of Acıbadem University (approval number: ATADEK-2024-2/67, Date: 15 February 2024).

**Figure 1:**
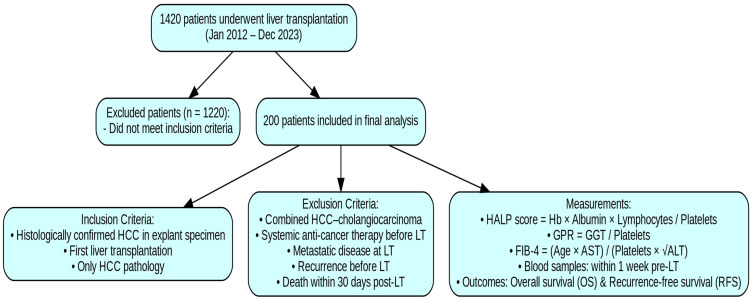
Flowchart of the study.

A total of 1420 patients underwent LT for various indications during this period. Among these, 200 patients met the predefined inclusion criteria and were enrolled in the final analysis. Inclusion criteria were as follows: (1) patients with histologically confirmed primary liver cancer in the explant specimen; (2) patients who underwent liver transplantation for the first time; and (3) patients whose explant pathology revealed only HCC, with no evidence of mixed tumors or additional malignancies. Exclusion criteria included the following: (1) the presence of combined hepatocellular-cholangiocarcinoma; (2) receipt of systemic anti-cancer therapy prior to transplantation; (3) evidence of metastatic disease at the time of LT; (4) recurrence of HCC prior to LT; and (5) death due to any cause within the first 30 days following LT (Figure 1).

### 2.2. Measurements

Pretransplant bridging therapy—including transarterial chemoembolization (TACE), radiofrequency ablation (RFA), or, in select cases, microwave ablation (MWA)—was administered to patients whose waiting time exceeded 3 months. No patients received systemic chemotherapy or selective internal radiotherapy in this cohort. Patients who initially did not meet transplantation criteria underwent downstaging (DS) protocols using similar locoregional therapies to reduce tumor burden and qualify for LT. An exploratory subgroup analysis was performed to assess whether pretransplant locoregional treatment influenced baseline HALP, GPR, and FIB-4 values or their prognostic associations with OS and RFS.

Peripheral venous blood samples were collected within one week prior to the transplant procedure. The median time interval between the initial diagnosis of HCC (radiologically or histopathologically confirmed) and the pre-transplant measurement of HALP, GPR, and FIB-4 was 5.5 months (IQR: 3.0–9.0 months), reflecting variations in individual waiting list durations. The following indices were calculated using standard laboratory values:

• **HALP score**: hemoglobin (g/L) × albumin (g/L) × lymphocyte count (10^9^/L)/platelet count (10^9^/L) [18].
• **GPR (gamma-glutamyl transpeptidase to platelet ratio)**: GGT (U/L)/platelet count (10^9^/L) [19].
• **FIB-4 index**: (age [years] × AST [U/L])/(platelet count [10^9^/L] × √ALT [U/L]) [20].
• **Outcomes:**
• In this study, HALP, GPR, and FIB-4 values were assessed only in the pre-transplant period. No standardized post-transplant measurement schedule was implemented for these indices; subsequent laboratory evaluations were performed based on clinical indications and were not uniformly collected across the cohort.

The median follow-up duration was calculated from the date of transplantation to either the last clinical follow-up or the patient’s death

### 2.3. Statistical Analysis

All statistical analyses were performed using SPSS version 27.0 (IBM Corp., Armonk, NY, USA), and all statistical analyses were conducted using IBM SPSS Statistics for Windows, version 27.0 (IBM Corp., Armonk, NY, USA). Continuous variables were presented as mean ± standard deviation (SD) or median with 95% confidence intervals (CI), depending on the data distribution. The Shapiro–Wilk test was used to assess normality. For comparisons between groups, the independent samples *t*-test was applied for normally distributed variables, while the Mann–Whitney U test was used for non-normally distributed variables. Receiver operating characteristic (ROC) curve analysis was employed to determine optimal cut-off values for HALP, GPR, and FIB-4 indices with respect to overall survival (OS) and recurrence-free survival (RFS). The Youden index was used to select thresholds that provided the best balance between sensitivity and specificity. To identify potential predictors of OS and RFS, univariate Cox proportional hazards regression analysis was performed. Variables with a *p*-value < 0.10 in univariate analysis were subsequently included in multivariate Cox regression models to determine independent prognostic factors. Hazard ratios (HRs) and 95% confidence intervals were calculated for each variable. Survival probabilities were estimated using the Kaplan–Meier method, and differences between groups were evaluated using the log-rank test. A *p*-value of less than 0.05 was considered statistically significant in all analyses.

## 3. Results

Comparison: The demographic and clinicopathological characteristics were shown in Table 1. Among the 200 patients included in the study, the majority were male (83.0%), with a median age of 66 years [IQR: 59–71]. The predominant etiologies of underlying liver disease were Hepatitis B Virus (56.5%) and cryptogenic cirrhosis (15.0%). The median BMI was 26 kg/m^2^ [IQR: 24–29]. According to Child–Pugh classification, 47.0% of patients were classified as A and 47.0% as B, while 6.0% had advanced liver dysfunction (Child–Pugh C). The median MELD score was 11.5 [IQR: 8–17]. Preoperative biomarker analysis revealed a median HALP score of 0.411 [0.269–0.596], FIB-4 index of 5.755 [3.6125–8.590], and GPR of 0.81 [0.47–1.41]. The median AFP level was 8.6 ng/mL [3.6–51]. Median tumor size was 25 mm [IQR: 16–40], and the median number of HCC lesions was two [IQR: 1–3]. Histologically, 49.5% of tumors had intermediate differentiation, while 33.0% were well-differentiated and 17.5% poorly differentiated. Microvascular invasion (MVI) was present in 37.5% of patients. Locoregional treatment prior to transplantation was applied in 32.5% of patients. During the follow-up period (median: 56 months [IQR: 31–80]), tumor recurrence was observed in 16.0% of patients, with a median recurrence-free survival of 45.5 months [IQR: 17–79]. Overall, 77.0% of the cohort died during follow-up. When comparing patients who received locoregional treatment (*n* = 65) with those who did not (*n* = 135), no statistically significant differences were observed in baseline HALP, GPR, or FIB-4 values (all *p* > 0.05). Moreover, the prognostic performance of these indices for OS and RFS remained consistent regardless of prior locoregional therapy status. Similarly, when patients were stratified according to the underlying liver disease etiology (e.g., HBV, HCV, alcoholic liver disease, NAFLD, cryptogenic), no significant differences were detected in baseline HALP, GPR, or FIB-4 values (all *p* > 0.05). Subgroup analyses further confirmed that the predictive accuracy of these indices for OS and RFS did not differ significantly across the different etiological categories (Table 1).

Univariate and Multivariate Analysis of Factors for OS are shown in Table 2. In univariate analysis, larger tumor size (HR: 1.028, 95% CI: 1.016–1.041, *p* < 0.001), higher number of HCC lesions (HR: 1.166, 95% CI: 1.088–1.250, *p* < 0.001), FIB-4 ≤ 3.1 (HR: 2.153, 95% CI: 1.161–3.994, *p* = 0.015), and GPR ≤ 0.45 (HR: 2.002, 95% CI: 1.100–3.645, *p* = 0.023) were significantly associated with poor overall survival. Although the HALP score ≤ 0.39 was not significant in univariate analysis (*p* = 0.171), it showed borderline association with survival. Multivariate Cox regression analysis confirmed that lower HALP scores (≤0.39) were independently associated with worse overall survival (HR: 0.480, 95% CI: 0.253–0.910, *p* = 0.024). Additionally, Child–Pugh C classification (HR: 4.588, 95% CI: 1.342–15.679, *p* = 0.015), tumor size (HR: 1.016, 95% CI: 1.003–1.030, *p* = 0.014), number of HCC lesions (HR: 1.157, 95% CI: 1.055–1.269, *p* = 0.002), FIB-4 ≤ 3.1 (HR: 2.467, 95% CI: 1.197–5.081, *p* = 0.014), and GPR ≤ 0.45 (HR: 2.438, 95% CI: 1.194–4.977, *p* = 0.014) were also found to be independent predictors of poor overall survival (Table 2).

Long- and short-term OS and RFS results for HALP, FIB-4, and GPR were shown in Table 3. Kaplan–Meier survival analysis demonstrated that lower FIB-4 (≤3.1) and GPR (≤0.45) scores were significantly associated with reduced overall survival (OS) at 1, 3, 5, and 10 years (*p* = 0.012 and *p* = 0.020, respectively). Specifically, patients with FIB-4 ≤ 3.1 had a 5-year OS rate of 66.5% compared to 83.3% in those with higher scores. Similarly, patients with GPR ≤ 0.45 had a 5-year OS of 58.9%, significantly lower than the 79.6% observed in those with GPR > 0.45. In contrast, the HALP score did not show a statistically significant difference in OS between groups (*p* = 0.165), despite a trend toward improved 5-year survival in patients with HALP ≤ 0.39 (79.7%).

In terms of recurrence-free survival (RFS), all three markers showed statistically significant associations. A lower HALP score (≤0.548) was significantly linked to reduced RFS at 1, 3, 5, and 10 years, with a 5-year RFS rate of 77.8% compared to 90.9% in patients with higher scores (*p* = 0.040). Similarly, patients with FIB-4 ≤ 7.88 and GPR ≤ 0.9 had significantly lower 5-year RFS rates (76.9% and 76.0%, respectively) compared to their counterparts with higher scores (91.8% and 96.2%, respectively; *p* = 0.028 and *p* = 0.047) (Table 3).

Univariate and Multivariate Analysis of Factors for RFS are shown in Table 4. Univariate analysis revealed that larger tumor size (HR: 1.028, 95% CI: 1.016–1.041, *p* < 0.001), higher number of HCC lesions (HR: 1.166, 95% CI: 1.088–1.250, *p* < 0.001), microvascular invasion (HR: 6.452, 95% CI: 2.789–14.921, *p* < 0.001), FIB-4 ≤ 7.88 (HR: 2.224, 95% CI: 0.915–5.406, *p* = 0.078), and GPR ≤ 0.9 (HR: 2.006, 95% CI: 0.990–4.061, *p* = 0.053) were associated with an increased risk of recurrence.

In multivariate analysis, the number of HCC lesions (HR: 1.142, 95% CI: 1.045–1.247, *p* = 0.003), tumor size (HR: 1.022, 95% CI: 1.006–1.037, *p* = 0.006), and microvascular invasion (HR: 3.423, 95% CI: 1.260–9.305, *p* = 0.016) remained significant independent predictors of recurrence. Furthermore, both FIB-4 ≤ 7.88 (HR: 3.952, 95% CI: 1.299–12.024, *p* = 0.015) and GPR ≤ 0.9 (HR: 2.582, 95% CI: 1.080–6.174, *p* = 0.033) were independently associated with poor recurrence-free survival, whereas HALP score ≤ 0.548 did not reach statistical significance (*p* = 0.143) (Table 4).

**Figure 2:**
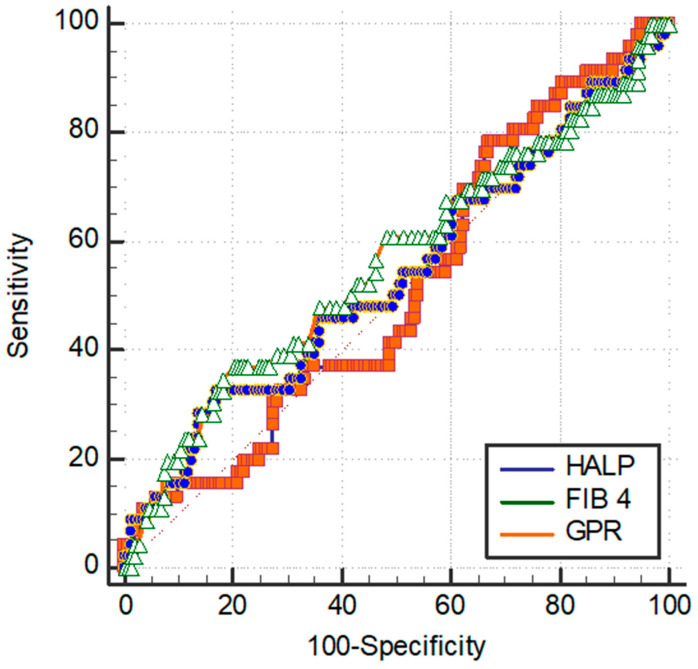
The ROC curves of GPR, FIB-4, and HALP score for overall survival.

The optimal cutoff values of GPR, FIB-4, and HALP scores for OS were 0.45, 3.1, and 0.39, respectively (Figure 2).

**Figure 3:**
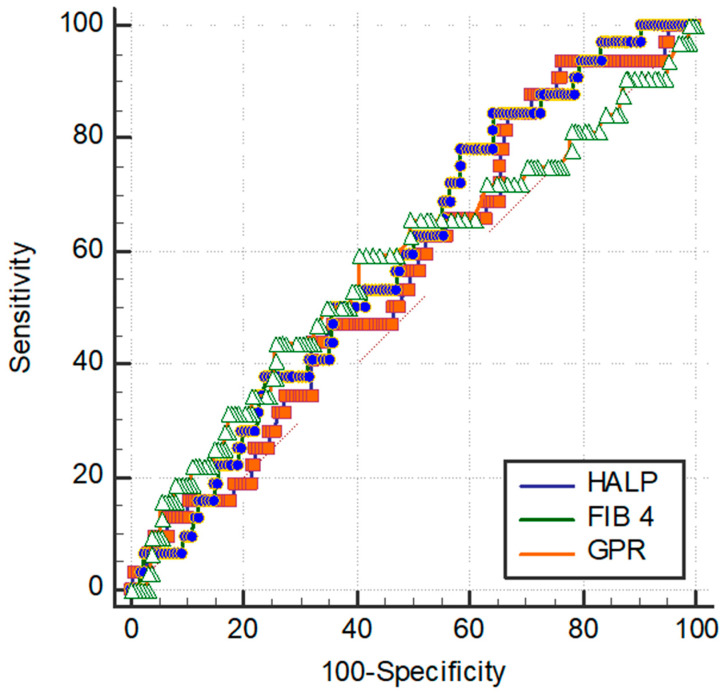
The ROC curves of GPR, Fib-4, and HALP score for tumor recurrence.

The optimal cut-off values of GPR, FIB-4, and HALP scores for tumor recurrence were 0.9, 7.88, and 0.54, respectively (Figure 3).

## 4. Discussion

In this retrospective cohort study involving liver transplant recipients with hepatocellular carcinoma (HCC), we demonstrated that preoperative inflammatory and immunonutritional indices, particularly the HALP score, FIB-4 index, and GPR, hold significant prognostic value for both overall survival (OS) and recurrence-free survival (RFS). A low HALP score (≤0.39) was independently associated with reduced OS but not with increased recurrence risk. In contrast, elevated FIB-4 (≤3.1) and GPR (≤0.45) values were predictive of both poor OS and higher recurrence rates. Tumor-related factors including larger tumor size, a greater number of HCC lesions, and the presence of microvascular invasion were also independently associated with worse prognosis. Collectively, these findings suggest that integrating readily available biomarkers such as HALP, FIB-4, and GPR with conventional morphologic features could enhance the stratification of HCC patients prior to liver transplantation and help identify individuals at higher risk for recurrence and mortality.

Previous studies had highlighted the prognostic utility of the HALP score in various malignancies, including hepatocellular carcinoma. Zhou et al. reported that a lower HALP score was associated with poor prognosis in HCC patients, particularly those who underwent resection [21]. Toshida et al. found the HALP score to be a significant predictor of long-term outcomes in Child–Pugh A patients who underwent curative resection for small HCC lesions [22]. Our findings extended these results to a transplant cohort and suggested that a low HALP score was also independently associated with poorer overall survival in patients who underwent liver transplantation. However, unlike prior studies that focused on resection, we found that HALP was not predictive of recurrence-free survival (RFS). This discrepancy might have been attributed to the distinct immune and regenerative environments following transplantation, in which systemic immunonutritional status influenced mortality risk more than local tumor behavior.

Our study also evaluated the prognostic roles of FIB-4 and GPR, two non-invasive indices that reflect hepatic inflammation and fibrosis. Yun et al. demonstrated that FIB-4 was a strong predictor of long-term survival in HCC patients who underwent curative hepatectomy, even in hepatitis B virus-dominant populations [20]. Consistent with this, our analysis identified low FIB-4 scores (≤3.1) as independent predictors of both OS and RFS in the liver transplant population. Moreover, GPR, which was shown by Wang et al. to correlate with liver fibrosis severity in chronic hepatitis B patients, also emerged as an independent prognostic factor for both OS and recurrence [12]. Yang et al. further supported the prognostic relevance of GPR in patients who underwent resection for solitary HCC lesions [19]; our findings expanded its utility to post-transplant outcomes and suggested that GPR might have served as a simple, cost-effective preoperative marker for recurrence risk stratification. Additionally, we found that the prognostic utility of HALP, GPR, and FIB-4 was consistent regardless of the underlying etiology of liver disease. This finding suggests that these biomarkers reflect systemic inflammatory and immunonutritional status rather than being substantially influenced by specific etiological factors such as viral hepatitis, alcohol-related liver disease, or NAFLD.

Microvascular invasion (MVI) remained a critical histopathological determinant of recurrence and poor prognosis in HCC. DiNorcia et al. and Qu et al. emphasized that MVI, although not preoperatively assessable, had the strongest association with post-transplant recurrence and should have been considered in risk models when explant histology was available [23,24]. In our study, MVI was an independent predictor of RFS but did not reach statistical significance for OS in multivariate analysis, possibly due to the dominant influence of systemic inflammatory markers such as HALP and FIB-4 on long-term survival. In addition, our results revealed that Child–Pugh C, tumor size, and number of HCC lesions were other factors that independently affected OS in HCC patients who underwent LT. These findings were consistent with previous studies [25]. We also found similar results to that reported in the previous studies; tumor size, number, and MVI were independent prognostic factors for recurrence and RFS [26,27].

Morphological parameters—namely tumor size and number—continued to play a crucial role in transplant eligibility and outcome prediction. Our results were in line with Mazzaferro et al. and subsequent validations of the Milan criteria, which established tumor burden as a primary determinant of recurrence and survival [28,29]. Although newer expanded criteria attempted to integrate biological behavior into selection algorithms, our data suggested that adding simple preoperative biomarkers like HALP, FIB-4, and GPR might have offered a practical enhancement to existing models without the need for complex molecular profiling.

Interestingly, our study found that patients with low FIB-4 and GPR scores had poorer survival and higher recurrence rates, despite these scores traditionally being associated with lower degrees of fibrosis. This paradox might have reflected the fact that liver transplantation eliminated cirrhotic tissue and altered disease dynamics, making systemic inflammation and immune status more influential than the degree of fibrosis per se. Garrido and Djouder previously questioned the assumption that cirrhosis was always a driver of HCC progression, suggesting that fibrotic remodeling might, in some cases, have restrained tumor spread by limiting vascular access [30]. In our cohort, these altered post-transplant dynamics might have explained why even low fibrosis markers were associated with poor outcomes.

Since the introduction of the Milan criteria in 1996, morphometric parameters such as tumor size and number have been widely adopted to guide candidate selection for liver transplantation and to mitigate the risk of post-transplant recurrence. Mazzaferro et al. confirmed the effectiveness of these criteria in selecting early-stage HCC patients with favorable outcomes [29]. Nevertheless, subsequent studies, including those by Mazzaferro et al. and Harper et al., revealed substantial inconsistencies—reporting up to 20–40% discrepancy between pretransplant imaging and explant histopathology—thereby questioning the adequacy of morphology-based models alone [28,31]. In our study, tumor size and number remained significant predictors of overall and recurrence-free survival, reaffirming their foundational role. However, the inclusion of biologically based indices—HALP, FIB-4, and GPR—offered an enhanced risk stratification framework, emphasizing their complementary value alongside morphologic parameters. To address the inherent limitations of radiologic assessment, DiNorcia et al. and Qu et al. proposed integrating histopathological markers such as microvascular invasion (MVI) and tumor differentiation grade into prognostic models [23,24]. Our data supported these suggestions, with MVI emerging as an independent predictor of recurrence. However, as also noted in the previous literature, MVI cannot be assessed preoperatively, which limits its practical utility in initial transplant decision-making. Serum-based biomarkers have gained increasing attention in this context. DiNorcia et al. noted the prognostic importance of alpha-fetoprotein (AFP) but also highlighted the inconsistencies in cut-off values among different clinical protocols [23]. Departing from this heterogeneity, our study focused on FIB-4 and GPR as more stable indicators of hepatic fibrosis and systemic inflammation. Both were independently associated with worse OS and RFS, suggesting their reliability and practicality in pretransplant evaluation. In addition, emerging research has drawn attention to the role of immune modulation in HCC prognosis. Heinrich et al. and Li et al. demonstrated that immune cell composition within the tumor microenvironment significantly influenced recurrence risk and long-term outcomes following transplantation [7,32]. In line with these observations, our study found the HALP score—an index that reflects immunonutritional status through lymphocyte and albumin levels—to be significantly associated with overall survival. This underscores the prognostic relevance of immune competence, further advocating for the integration of immune-inflammatory markers into future transplant selection criteria.

Although various methods—including liver biopsy, laboratory tests, and imaging—have been employed to assess the degree of fibrosis in cirrhotic patients with HCC, widely accepted clinical staging systems such as the Child–Pugh (CP) score and the Barcelona Clinic Liver Cancer (BCLC) classification had been predominantly used to evaluate liver function and guide treatment strategies [33,34]. However, these models relied on semi-quantitative and subjective variables like ascites and hepatic encephalopathy, which limited their precision in predicting long-term outcomes, particularly in the transplant setting. In contrast, non-invasive indices such as the FIB-4 score and gamma-glutamyl transpeptidase to platelet ratio (GPR) offered quantifiable surrogates of hepatic fibrosis and inflammation, yet their prognostic roles in post-LT patients had not been clearly established in the earlier literature. In our study, we evaluated the prognostic significance of these indices in liver transplant recipients and found that a low preoperative GPR was significantly associated with worse overall survival and a higher recurrence risk. Moreover, GPR emerged as an independent predictor of poor recurrence-free survival, suggesting its potential utility in identifying high-risk patients before transplantation. These findings partially diverged from those of Wang et al. and Zhang et al., who reported that lower GPR values were associated with better prognosis in non-transplant HCC cohorts [11,12]. However, their studies were limited by smaller sample sizes and focused solely on preoperative patients, which may have accounted for the observed differences. Yang et al. also emphasized the prognostic relevance of GPR following resection of solitary HCC lesions but did not assess its predictive value in transplant recipients [19]. Our results expanded upon these earlier findings by demonstrating that GPR retained prognostic value in the unique immunological and hemodynamic context of liver transplantation.

Yokomori et al. had proposed that structural distortion and impaired lymphatic drainage in cirrhotic livers might reduce the dissemination of tumor cells to lymph nodes, potentially altering metastatic behavior [35]. This hypothesis could partly explain the paradoxical association we observed between low GPR and poor outcomes, suggesting that systemic inflammation, rather than fibrosis alone, may have exerted a stronger influence on post-transplant prognosis. Overall, our findings underscored the value of incorporating reproducible, non-invasive markers such as GPR into pretransplant evaluation protocols to enhance risk stratification and individualized clinical planning.

Compared to previously published studies, our research provided several distinct contributions. Most prior investigations that evaluated the prognostic value of HALP, FIB-4, and GPR in HCC primarily focused on patients who underwent hepatic resection or received non-transplant interventions such as transarterial chemoembolization (TACE) [20,21,22]. In contrast, our study was among the few that examined the predictive utility of these markers specifically in the context of liver transplantation, where the immune environment and hepatic dynamics were substantially altered following surgery. Furthermore, earlier studies—such as those by Zhou et al. and Yang et al.—reported associations between low HALP or high GPR values and poor survival in surgical cohorts but did not assess recurrence-free survival (RFS) in transplant recipients [19,21]. Our study not only confirmed the prognostic relevance of these indices for overall survival but also demonstrated their independent associations with recurrence, particularly for FIB-4 and GPR, in the transplant setting. Importantly, while HALP had previously been validated mainly in patients with well-preserved liver function (e.g., Child–Pugh A), we included a broader range of liver dysfunction (Child–Pugh A to C), which expanded the applicability of our findings. By establishing separate ROC-based cut-off values for both OS and RFS, our study also introduced clinically useful thresholds tailored specifically for liver transplant patients—an aspect that earlier studies had not addressed. These methodological and cohort-based differences underscored the novelty and clinical relevance of our research in refining pretransplant risk stratification.

### Limitations of the Study

The study has some limitations. Firstly, there is a risk of bias due to its retrospective nature. Studies examining HALP have generally had a wide heterogeneity in the affected populations and depend on the threshold value of HALP. Each study used one of three different methods to determine the optimal HALP threshold. The most commonly used methods are X-tile software (version 3.6.1; Yale University, New Haven, CT, USA), ROC curves, and the median/mean approach. We also used the ROC curve method. We established separate cut-off values for OS and recurrence, and completed the analysis accordingly. There is no generally accepted threshold value determined in healthy populations, which inherently limits the use of HALP. Additionally, the use of HALP may be restricted in cancer patients with certain comorbidities. The markers included in HALP can mostly be influenced by comorbidity factors. Therefore, while it is a good prognostic indicator, it is unclear whether HALP can be used equally in patients with hepatocellular carcinoma who have different comorbidities. In our study, we did not conduct a separate subgroup analysis regarding this. There is a need for prospective randomized studies to definitively determine whether HALP is effective in these patients. Furthermore, in our study HALP, GPR, and FIB-4 values were evaluated only in the pre-transplant period. These indices were not re-assessed at standardized time points following liver transplantation, and post-transplant measurements were performed solely based on clinical indications. Therefore, potential changes in these markers over time and their dynamic prognostic value after transplantation could not be evaluated.

## 5. Conclusions

In conclusion, this study demonstrated that the preoperative HALP score served as an independent predictor of overall survival in patients with hepatocellular carcinoma undergoing liver transplantation. Although HALP was not significantly associated with recurrence or recurrence-free survival (RFS), its prognostic value for long-term survival was evident. More notably, both GPR and FIB-4 indices emerged as robust predictors for overall survival, recurrence risk, and RFS, underscoring their clinical utility in the pretransplant setting. The incorporation of these easily obtainable, cost-effective biomarkers into routine assessment protocols could enhance individualized risk stratification, guide clinical decision-making, and ultimately improve post-transplant outcomes in HCC patients. These findings highlight the potential of immunonutritional and inflammatory indices to complement traditional morphologic criteria in modern transplant oncology.

## Figures and Tables

**Table 1:** Demographics and Clinical-pathological characteristics of HCC patients.

**Gender**	Male	166 (83.0)
	Female	34 (17.0)
**Age** Median [IQR]		66 [59–71]
**BMI** Median [IQR]		26 [24–29]
**Diagnosis**	Hepatitis B Virus	113 (56.5)
	Cryptogenic	30 (15.0)
	Hepatitis C Virus	26 (13.0)
	Ethanol	12 (6.0)
	NASH	7 (3.5)
	Budd-Chiari	3 (1.5)
	Autoimmune	2 (1.0)
	HCC	2 (1.0)
	Wilson	2 (1.0)
	Alcohol	1 (0.5)
	Hemochromatosis	1 (0.5)
	Cholestasis	1 (0.5)
**Child–Pugh**	A	94 (47.0)
	B	94 (47.0)
	C	12 (6.0)
**MELD** Median [IQR]		11.5 [8–17]
**AFP** Median [IQR]		8.6 [3.6–51]
**HALP** Median [IQR]		0.411 [0.269–0.596]
**FIB-4** Median [IQR]		5.755 [3.6125–8.590]
**GPR** Median [IQR]		0.81 [0.47–1.41]
**Tumor size (mm)** Median [IQR]		25 [16–40]
**Number of HCC lesions** Median [IQR]		2 [1–3]
**Tumor differentiation**	Intermediate	99 (49.5)
	Early	66 (33.0)
	Advanced	35 (17.5)
**Microvascular invasion**	No	125 (62.5)
	Yes	75 (37.5)
**Locoregional treatment**	No	135 (67.5)
	Yes	65 (32.5)
**Tumor recurrence**	No	168 (84.0)
	Yes	32 (16.0)
**Recurrence free time** Median [IQR]		45.5 [17–79]
**Follow up time** Median [IQR]		56 [31.25–80]
**Died**	Yes	154 (77.0)
	No	46 (23.0)

BMI: Body mass index, AFP: Alpha fetoprotein, HALP: Hemoglobin, Albumin, Lymphocyte, Platelet, FIB-4: Fibrosis-4 score, GPR: Gamma glutamyl transpeptidase to platelet ratio, HCC: Hepatocellular carcinoma, and NASH: Non-Alcoholic Steatohepatitis.

**Table 2:** Univariate and Multivariate Analysis of Factors for OS.

	Univariate				Multivariate			
			95% CI for HR				95% CI for HR	
	*p*	HR	Lower	Upper	*p*	HR	Lower	Upper
Gender (Ref: Female) Male	0.765	0.873	0.359	2.122	0.410	1.453	0.597	3.536
Age	0.360	0.982	0.944	1.021	0.063	1.039	0.998	1.082
BMI	0.513	0.967	0.876	1.069	0.303	1.048	0.959	1.144
Child–Pugh (Ref: A)	0.619				0.043			
Child–Pugh B	0.605	0.824	0.396	1.714	0.114	1.826	0.866	3.852
Child–Pugh C	0.507	1.519	0.442	5.212	0.015	4.588	1.342	15.679
MELD	0.540	0.983	0.930	1.039	0.885	1.004	0.948	1.064
AFP	0.774	1.000	0.999	1.001	0.824	1.000	0.999	1.001
Tumor size (mm)	<0.001	1.028	1.016	1.041	0.014	1.016	1.003	1.030
Number of HCC lesions	<0.001	1.166	1.088	1.250	0.002	1.157	1.055	1.269
Local regional treatment	0.078	1.869	0.933	3.744	0.746	0.892	0.447	1.779
Tumor differentiation (Ref: intermediate)	0.233				0.720			
Advance	0.283	1.565	0.691	3.541	0.650	1.210	0.531	2.754
Early	0.371	0.667	0.274	1.620	0.596	0.794	0.338	1.866
Microvascular invasion	<0.001	6.452	2.789	14.921	0.421	1.361	0.642	2.885
HALP (Ref: >0.39) ≤ 0.39	0.171	0.658	0.362	1.198	0.024	0.480	0.253	0.910
FIB-4 (Ref: >3.1) ≤ 3.1	0.015	2.153	1.161	3.994	0.014	2.467	1.197	5.081
GPR (Ref: >0.45) ≤ 0.45	0.023	2.002	1.100	3.645	0.014	2.438	1.194	4.977

BMI: Body mass index, AFP: Alpha fetoprotein, HALP: Hemoglobin, Albumin, Lymphocyte, Platelet, FIB-4: Fibrosis-4 score, and GPR: Gamma glutamyl transpeptidase to platelet ratio.

**Table 3:** Long- and short-term OS and RFS results for HALP, FIB-4, and GPR.

		OS				
		1 Year	3 Years	5 Years	10 Years	Log Rank *p*
**HALP**	>0.39	%86.8 (SE:3.3)	%76.8 (SE:4.2)	%70.4 (SE:4.7)	%70.4 (SE:4.7)	0.165
	≤0.39	%87.8 (SE:3.5)	%84.4 (SE:3.8)	%79.7 (SE:4.5)	%79.7 (SE:4.5)	
**FIB-4**	>3.1	%87.8 (SE:2.6)	%83.8 (SE:3.0)	%78.8 (SE:3.4)	%78.8 (SE:3.4)	0.012
	≤3.1	%85.2 (SE:5.6)	%66.5 (SE:7.6)	%58.4 (SE:8.6)	-	
**GPR**	>0.45	%87.9 (SE:2.7)	%82.3 (SE:3.2)	%79.6 (SE:3.4)	%79.6 (SE:3.4)	0.020
	≤0.45	%85.4 (SE:5.1)	%74.3 (SE:6.4)	%58.9 (SE:8.0)	%58.9 (SE:8.0)	
		**RFS**				
		**1 year**	**3 years**	**5 years**	**10 years**	
**HALP**	>0.548	%96.4 (SE:2.5)	%90.9 (SE:3.9)	%90.9 (SE:3.9)	%90.9 (SE:3.9)	0.040
	≤0.548	%88.5 (SE:2.8)	%79.0 (SE:3.7)	%77.8 (SE:3.8)	%77.8 (SE:3.8)	
**FIB-4**	>7.88	%93.5 (SE:3.1)	%91.8 (SE:3.5)	%91.8 (SE:3.5)	%91.8 (SE:3.5)	0.028
	≤7.88	%89.6 (SE:2.7)	%78.1 (SE:3.8)	%76.9 (SE:4.0)	%76.9 (SE:4.0)	
**GPR**	>0.9	%94.2 (SE:2.3)	%87.8 (SE:3.3)	%86.2 (SE:3.6)	%86.2 (SE:3.6)	0.047
	≤0.9	%86.7 (SE:3.7)	%76.0 (SE:4.8)	%76.0 (SE:4.8)	%76.0 (SE:4.8)	

HALP: Hemoglobin, albumin, lymphocyte, platelet, FIB-4: Fibrosis-4 score, GPR: gamma glutamyl transpeptidase to platelet ratio, OS: Overall survival, and RFS: recurrence free survival.

**Table 4:** Univariate and Multivariate Analysis of Factors for RFS.

	Univariate				Multivariate			
	*p*	HR	95% CI for HR		*p*	HR	95% CI for HR	
			Lower	Upper			Lower	Upper
**Gender** (Ref: Female) Male	0.765	0.873	0.359	2.122	0.472	1.557	0.466	5.199
**Age**	0.360	0.982	0.944	1.021	0.375	1.022	0.974	1.072
**BMI**	0.513	0.967	0.876	1.069	0.315	0.940	0.833	1.061
**Child–Pugh** (Ref: A)	0.619				0.599			
Child–Pugh B	0.605	0.824	0.396	1.714	0.732	1.177	0.463	2.993
Child–Pugh C	0.507	1.519	0.442	5.212	0.313	2.267	0.462	11.127
**MELD**	0.540	0.983	0.930	1.039	0.589	1.023	0.942	1.110
**AFP**	0.774	1.000	0.999	1.001	0.797	1.000	0.998	1.001
**Tumor size mm**	<0.001	1.028	1.016	1.041	0.006	1.022	1.006	1.037
**Number of HCC lesions**	<0.001	1.166	1.088	1.250	0.003	1.142	1.045	1.247
**Local regional treatment**	0.078	1.869	0.933	3.744	0.666	1.200	0.525	2.741
**Tumor differentiation** (Ref: intermediate)	0.233				0.986			
Advance	0.283	1.565	0.691	3.541	0.985	1.009	0.392	2.597
Early	0.371	0.667	0.274	1.620	0.872	0.921	0.336	2.522
**Microvascular invasion**	<0.001	6.452	2.789	14.921	0.016	3.423	1.260	9.305
**HALP** (Ref: >0.548) ≤0.548	0.104	2.090	0.860	5.078	0.143	2.078	0.780	5.532
**FIB-4** (Ref: >7.88) ≤7.88	0.078	2.224	0.915	5.406	0.015	3.952	1.299	12.024
**GPR** (Ref: >0.9) ≤ 0.9	0.053	2.006	0.990	4.061	0.033	2.582	1.080	6.174

## Data Availability

Data are available upon request to the corresponding author.
